# A novel method for furfural recovery via gas stripping assisted vapor permeation by a polydimethylsiloxane membrane

**DOI:** 10.1038/srep09428

**Published:** 2015-03-30

**Authors:** Song Hu, Yu Guan, Di Cai, Shufeng Li, Peiyong Qin, M. Nazmul Karim, Tianwei Tan

**Affiliations:** 1 National energy R&D center for biorefinery, Beijing University of Chemical Technology, Beijing, China; 2 Artie McFerrin Department of Chemical Engineering, Texas A&M University, College Station, USA

## Abstract

Furfural is an important platform chemical with a wide range of applications. However, due to the low concentration of furfural in the hydrolysate, the conventional methods for furfural recovery are energy-intensive and environmentally unfriendly. Considering the disadvantages of pervaporation (PV) and distillation in furfural separation, a novel energy-efficient ‘green technique’, gas stripping assisted vapor permeation (GSVP), was introduced in this work. In this process, the polydimethylsiloxane (PDMS) membrane was prepared by employing water as solvent. Coking in pipe and membrane fouling was virtually non-existent in this new process. In addition, GSVP was found to achieve the highest pervaporation separation index of 216200 (permeate concentration of 71.1 wt% and furfural flux of 4.09 kgm^−2^h^−1^) so far, which was approximately 2.5 times higher than that found in pervaporation at 95°C for recovering 6.0 wt% furfural from water. Moreover, the evaporation energy required for GSVP decreased by 35% to 44% relative to that of PV process. Finally, GSVP also displayed more promising potential in industrial application than PV, especially when coupled with the hydrolysis process or fermentation in biorefinery industry.

With the rapidly decreasing reserves of the world's fossil-fuel reserves, the need for developing renewable fuels and chemicals is becoming increasingly urgent. Among the numerous chemicals derived from biomass, furfural (2-furaldehyde) is an important platform chemical with a wide range of applications^1^,^2^. For instance, it can be directly used as solvent for refining lubricants and diesel oil^3^. Moreover, furfural is also employed as raw materials for manufacturing of furfuryl alcohol, tetrahydrofuran (THF), and resins^4^. Since there is no synthetic route available for furfural production, furfural only can be obtained from lignocellulosic biomass by dehydrating pentoses (mainly xylose)^5^,^6^.

In most industrial processes, the hemicellulose is converted into xylose by sulfuric acid catalyzed hydrolysis, and subsequently xylose is further dehydrated into furfural^7^,^8^. Both sets of reactions take place in the digester, which is maintained at high temperature by injecting steam^9^. Meanwhile, furfural is continuously extracted from the mixed solution by steam and then fed into distillation columns for purification^10^,^11^. However, the furfural yields are only between 45% and 50% due to its side reactions of high temperatures^12^. Moreover, large consumption of steam (about 20 tons steam per ton of furfural)^13^ also causes a significant waste of energy and a huge amount of wastewater. Besides, corrosion, serious coking, and high purification costs (caused by the low concentration of furfural in steam) were existed in the subsequent distillation process^14^. These drawbacks undoubtedly cause threatens to the environment and health of operations staff. Therefore, it is highly desirable to develop a highly efficient green separation technology that can in situ remove furfural from dilute aqueous solutions in a timely manner^15^.

Over the last decade, several methods—such as adsorption^16^, extraction^17^, and membrane separation^18^,^19^—have been investigated for furfural recovery. Among these, pervaporation (PV) is widely recognized as an energy-saving, low-cost and environmental friendly technique, and it performs satisfactorily in the separation of low concentration furfural/water system. In our previous work^20^, we have proved that pervaporation with the PDMS membrane (using water as a solvent) was a promising way for the separation of furfural. The process exhibited high separation performance (permeate concentration of 62.4 wt% and furfural flux of 3222.6 gm^−2^h^−1^) and excellent stability at 95°C for separating 6.5 wt% furfural solutions. Furthermore, the energy consumption of evaporation for pervaporation was at least 70% less than that for distillation. Nevertheless, all these studies were based on using furfural/water binary solution for separation; in the real reaction system during furfural production, there exists a high solid-to-liquid ratio (about 1:2 or 1:3) in the reaction solution. So a pretreatment (microfiltration) was needed before PV process in order to avoid fouling and damage of the pervaporation membrane. But the microfiltration membrane fouling was another problem.

In order to overcome these disadvantages and further improve the separation performance, we studied a new separation technique that integrated gas stripping and vapor permeation (GSVP). Its schematic diagram was shown in Fig. 1B. In the process, the air in the reactor was used as the carrier gas and circularly bubbled into the reaction solution so as to extract furfural from the liquid; the furfural in the gas mixture was then recovered by a PDMS membrane. The difference of process between GSVP and PV (Fig. 1A) was that gas mixture was fed to membrane in GSVP process while liquid solution was fed to membrane in PV process. For this method, there was no need to introduce any steam and no energy was consumed for vapour condensation in the feed side. Due to the use of gas for feeding in GSVP process, the pretreatment of reaction solutions wasn't required, and the coupling process of furfural production with in situ recovery was simplified. In our current work, the PDMS membrane was prepared by using water as the solvent in a green method. The contrast experiments between PV and GSVP for furfural recovery at different temperatures and feed concentrations were conducted, and then the performances were compared with those in the literature. The evaporation energy consumption of PV and GSVP were also compared. Finally, potential of GSVP in practical application for furfural recovery was pointed out and analysed.

## Methods

PDMS was obtained from Shandong Dayi Chemical Co., Ltd. PVDF membrane was prepared in our laboratory. Dodecyl benzene sulfonic acid (DBSA) was purchased from Nanjing Taijia Washing Chemical Co., Ltd., China. Furfural ≥ 99.0% and dibutyltin dilaurate (DBTDL) were purchased from Tianjing Fuchen Chemical Co., Ltd. Tetraethyl orthosilicate (TEOS) was obtained from Guangdong Xilong Chemical Co., Ltd. All these agents were used without further purification in this study.

The PDMS membranes were prepared on the PVDF membrane by employing water as the solvent in the presence of dodecyl benzene sulphonic acid (DBSA). The details can be seen in our previous work^20^,^21^.

The SEM (Scanning Electron Microscope) images of PDMS membrane were performed on a SU1510 scanning electron microscope (Hitachi High-Technologies Corporation, Japan). The thickness of the PDMS layer was measured from the cross-sectional image.

**Figure 1:**
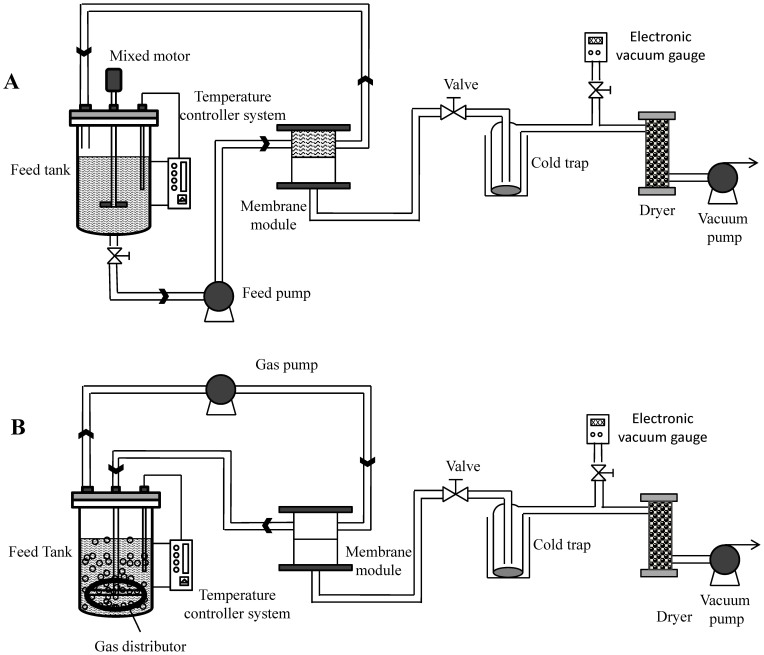
Schemes of the processes of (A) pervaporation and (B) gas stripping assisted vapor permeation.

The experiments dealing with PV and GSVP for furfural recovery were studied by using a lab-scale apparatus shown in Fig. 1A and Fig. 1B, respectively. The feed mixtures (2.5 L) with a composition of 1.0–6.0 wt% furfural were kept in the 3 L feed tanks. In PV experiments, the feed flow rate was about 0.3 Lmin^−1^ and the stirring speed was 150 rpm. In GSVP experiments, the gas circulation between the feed tank and the membrane module was carried out by a mini gas pump (PCF5015N, Chengdu Qihai E&M Manufacturing Co., Ltd, China) and the gas flow rate was about 10 Lmin^−1^ (optimized option, see Supplementary Fig. S2 online). The effective areas of membranes in both modules were 28.26 cm^2^. The downstream pressure of the membranes was maintained below 200 Pa by vacuum pumps (2XZ-2, Shanghai Deying Vacuum Lighting Equipment Co., Ltd., China) and was measured by SUMMIT-605 digital vacuum gauges (SUMMIT, Korea). The permeate vapors were condensed in cold traps cooled by liquid nitrogen and subsequently weighed every 1 h. The concentration of the feed solution and the condensate were analyzed by a GC-14C gas chromatograph equipped with a Porapak-Q packed column. To minimize the experimental error, all the experiments were repeated 2 times after the whole system reached a steady state (about 1 hour later).

The separation performance of both processes were evaluated by total flux (*J*), organic flux (*J_i_*), separation factor (*α*), and pervaporation separation index (PSI), which are calculated by the following equations: 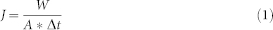

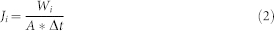
Here, *W* refers to the weight of the condensate in the cold trap; Δ*t* is the operating time; *A* is the effective area of the membrane; *W_i_* is the weight of furfural in the condensate.

In PV process, 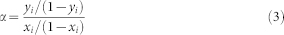
In GSVP process, the separation factor *α* of the whole process consists of gas stripping separation factor (*α_Strip_*) and membrane separation factor (*α_memb_*),
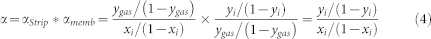

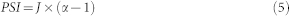
where *x_i_* and *y_i_* represent the mass ratio of furfural and water in the feed solution and permeate, respectively; *y_gas_* is the mass fraction of furfural in the condensate of gas mixtures in the feed side.

## Results and Discussion

### Characterization of the membrane

**Figure 2:**
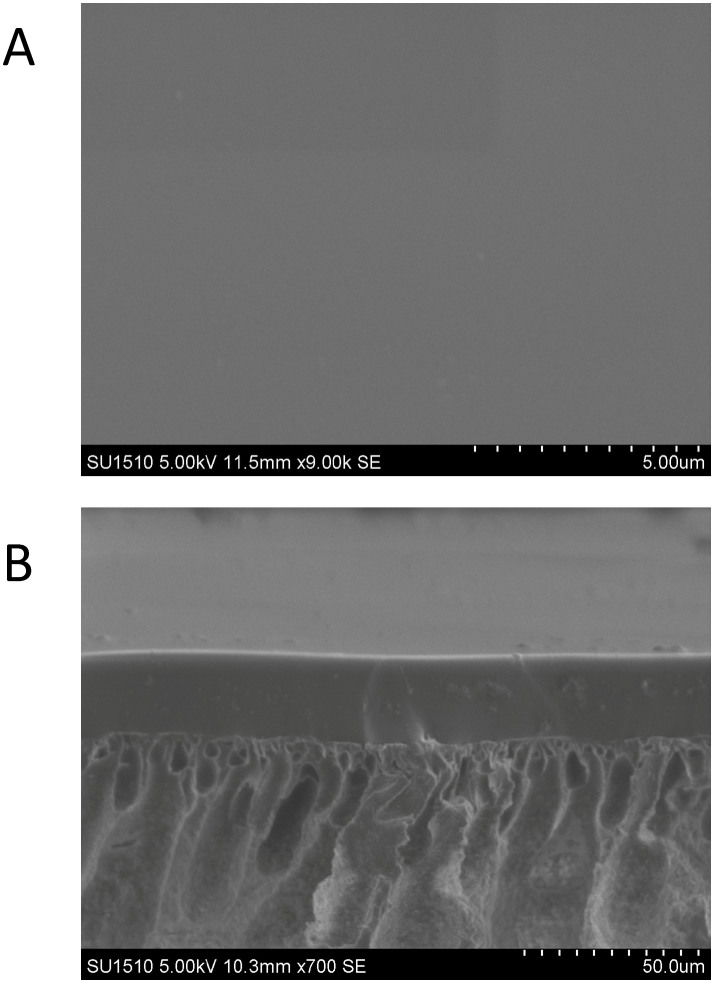
SEM images of (A) the surface and (B) cross-section of PDMS membrane prepared by using water as solvent.

As the Fig. 2 shows, the morphologies of the PDMS membrane prepared by the green method were observed. The membrane is composed of two layers as seen in the cross-sectional image (Fig. 2. B). The top layer is the PDMS layer (about 25 µm), which plays the main role of separation in the process. The bottom layer is polyvinylidene fluoride (PVDF) membrane that can prevent the casting solution leaking into the supports during membrane preparation (by appropriate surface pores) and provides good mechanical properties for the whole membrane.

Furthermore, in the process of PDMS membrane preparation, water was used as solvent to instead of organic solvent. For this method, the usage of large amount of organic solvent was avoided, and the investment in the aspect of explosion proofing and solvent recovery was decreased.

### Removal of furfural polymers and prevention of coking

Furfural is colorless, but it tends to undergo autoxidation and then polymerized gradually, and becomes a dark-colored substance during storage^9^,^22^. If the polymers are not removed in time, the concentration of the furfural polymer increases continuously until the furfural becomes unusable. Besides, the formation of these polymers in the process of furfural production is more rapid due to the high reaction temperature^9^. And the polymers coat on the surface of the equipment, causing corrosion and coking during the heating process, plugging and interfering with the heat transfer^23^. So it is necessary to find a way to overcome these disadvantages.

**Figure 3:**
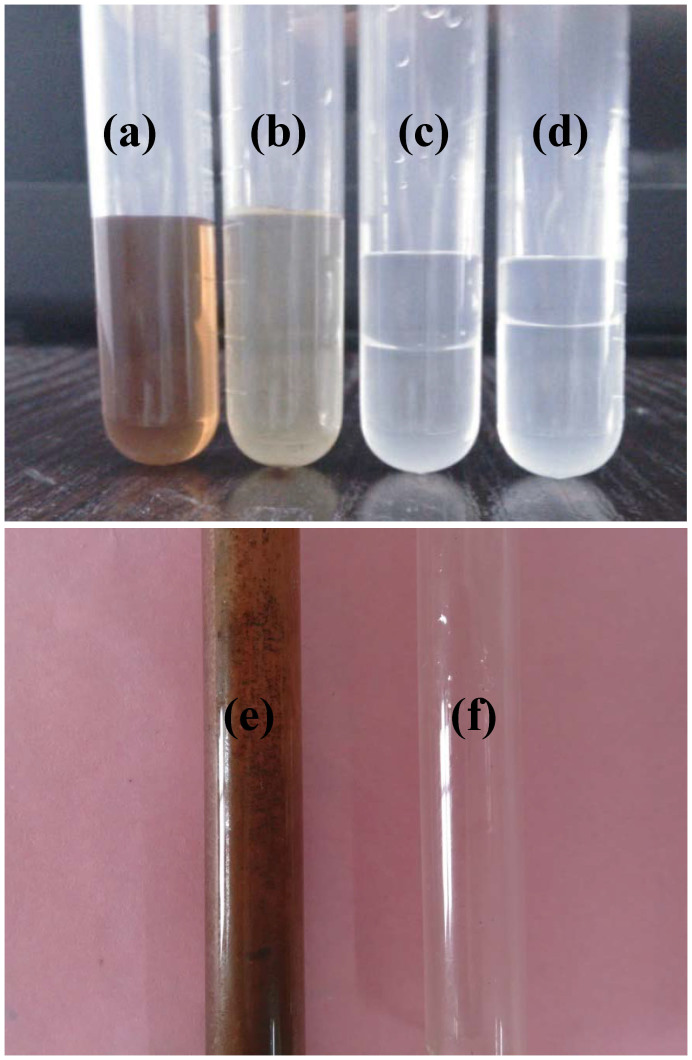
Images of (a) commercial furfural after aging a period of time, (b) feed mixture (containing 3 wt% furfural), (c) permeate in PV, (d) permeate in GSVP, and the glass pipelines in feed side of (e) PV process and (f) GSVP process.

In this study, PV and GSVP were applied in separating the mixture—comprised of furfural, water, furfural polymers, and some impurities. The mixture solution was obtained by aging commercial furfural for a period of time and then dissolving into water. The separation products and feed solution was shown in Fig. 3. As can be seen from Fig. 3(c) and Fig. 3(d), the permeates in PV and GSVP experiments showed two phases due to the high furfural concentration in water, and both phases were colorless. The results suggested that the chromogenic component (furfural polymer) in the feed solution was rejected by the PDMS membrane in the permeation process. It implied that PV and GSVP can remove furfural polymers and recover furfural from water at the same time, which is difficult to achieve by a distillation process^24^. Besides, it is evident from the Fig. 3(c) and Fig. 3(d) that the volume of the bottom phase (furfural concentration is 95.2 wt% at 20°C) in the condensate obtained in GSVP process was more than that in PV process, and smaller volume of top phase (furfural concentration is 8.3 wt% at 20°C) was obtained in the condensate in GSVP process. This phenomenon implied that GSVP showed better performance characteristic for removal of furfural from water.

**Figure 4:**
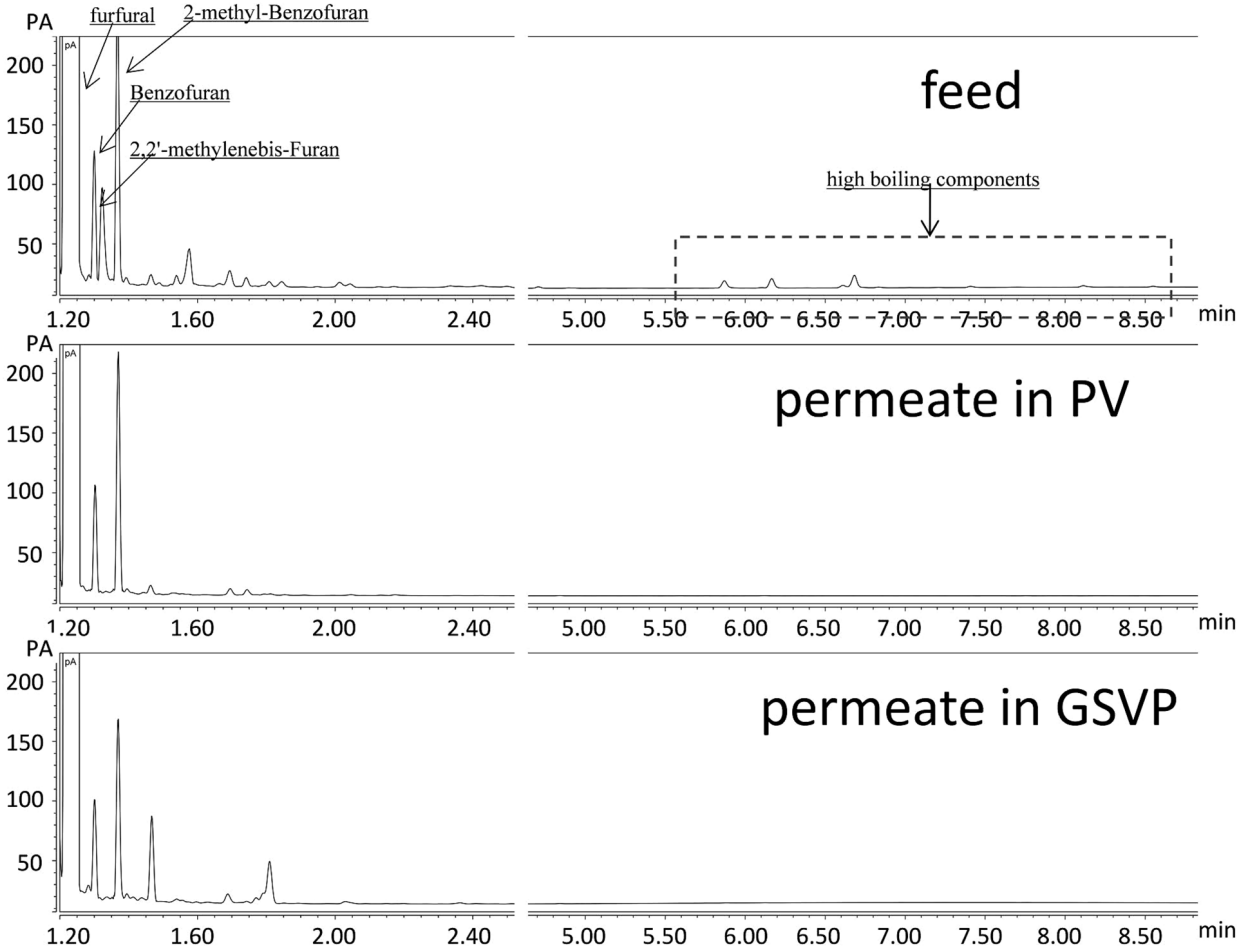
Comparisons of gas chromatographic charts of feed, permeate in PV, and permeate in GSVP.

The bottom phase (furfural) of both the condensates and the commercial furfural containing small percentages of furfural polymers were dissolved in ethyl acetate and then detected by a gas chromatograph (Trace 1300 GC, Thermo Scientific, USA) equipped with a hydrophobic capillary column (DB-1HT, 30 m, 0.32 mm). As it is seen in Fig. 4, high boiling components (mainly furfural polymers) in feed were not detected in the permeates of PV and GSVP. This means these components can be rejected by the PDMS membrane, which may be due to their higher molecular diameter resulting in high mass-transfer resistance through the PDMS membrane. However, some low boiling impurities in feed were also enriched in the permeate of GSVP due to the higher selectivity of GSVP for these organics, but the concentrations of these impurities were very low (with the perecent of peak areas less than 0.06%), which won't affect the property of the products.

After 2 days of operation of PV and GSVP apparatus, the glass pipelines in feed side are shown in Fig. 3(e) and Fig. 3(f), respectively. The phenomenon of coking on the glass pipeline in PV process was very serious. By contrary, this phenomenon was not encountered in GSVP process, which because the furfural polymers were non-volatile and so they could not contact with the pipelines in GSVP process. This result suggested that the problem of furfural polymers adhering and caking on the surface of components (such as membrane module and pipeline) could be solved by using gas as the feed in GSVP process. Therefore, the corrosion and blockage that caused by coke formation, which seriously hinder the industrial application of furfural production, also could be avoided.

All these experiments were performed by using furfural/water binary solution for separation. However, in furfural production, there is a large amount of solid residue in the reaction solution. So a pretreatment (microfiltration) was needed before PV process in order to avoid fouling and damage of the pervaporation membrane. But the problem of microfiltration membrane fouling has still not been solved. Fortunately, these problems are not encountered in GSVP process due to the use of gas for feeding; and no any pretreatment was needed before the GSVP process.

### A comparison of separation performance between PV and GSVP

**Figure 5:**
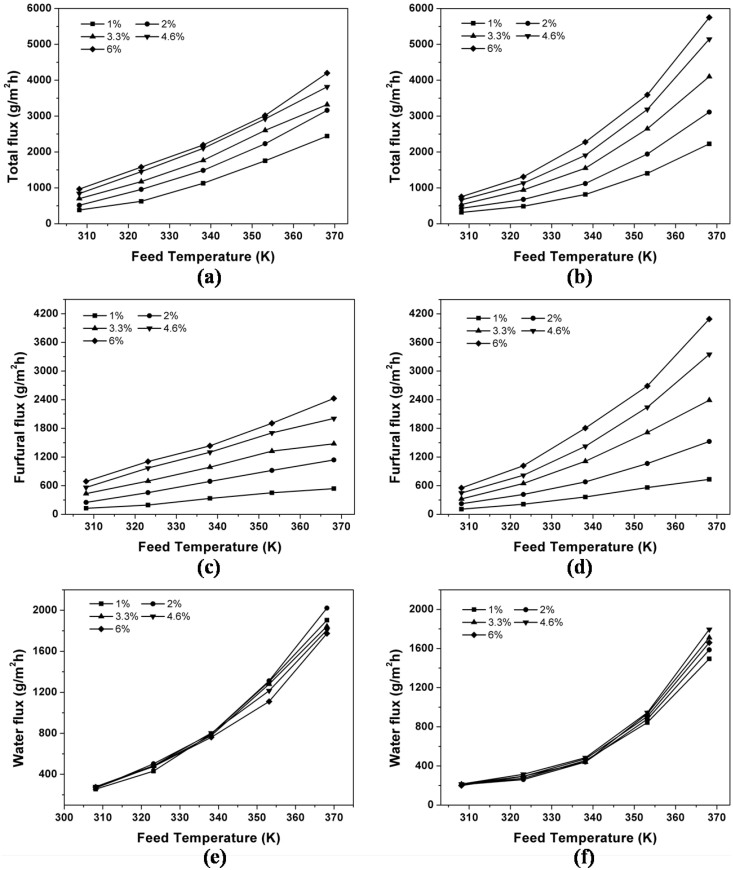
Effect of feed temperature on the total flux of (a) PV and (b) GSVP, the furfural flux of (c) PV and (d) GSVP, and the water flux of (e) PV and (f) GSVP.

**Figure 6:**
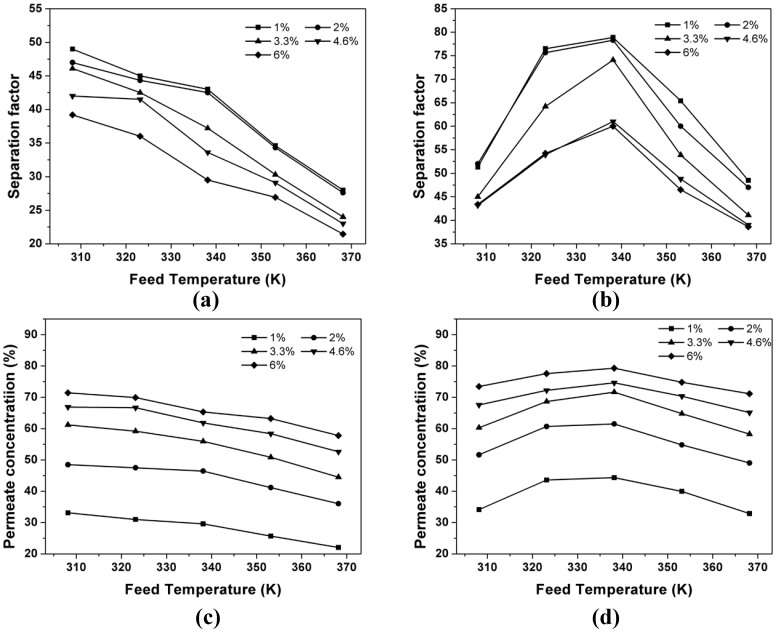
Effect of feed temperature on the separation factor of (a) PV and (b) GSVP and the permeate concentration in (c) PV and (d) GSVP.

In furfural production, the reactions during the hydrolysis of hemicelluloses and further dehydration are achieved at high temperatures (more than 90°C). Therefore, the separation performance of PV and GSVP at high operating temperature should be considered. To compare the separation performance between GSVP and PV, especially at high temperatures, experiments were carried out as follows. PV and GSVP experiments were conducted at different furfural concentrations (from 1.0 wt% to 6.0 wt%) and feed temperatures (from 308.15 K to 368.15 K) by the same membrane. The total flux, furfural flux, separation factor, and permeate concentration of both the processes are calculated and plotted in Fig. 5 and Fig. 6 for comparison.

As can be seen from Fig. 5(c) – (d), GSVP possessed higher furfural permeate flux for furfural recovery, especially at high temperatures and high feed concentrations. For example, the furfural permeate flux in GSVP process reached 4090 gm^−2^h^−1^ for separating 6 wt% furfural/water solution at 95°C, while the furfural permeate flux in PV process only attained 2426 gm^−2^h^−1^. This may be caused by the temperature polarization and concentration polarization, which are often appear in PV process with a high permeability and selectivity membrane but not exist in vapor permeation process^25^,^26^. The effects of these phenomena are intensified with an increase in membrane permeate flux^27^,^28^. So the fluxes of both processes showed in Fig. 5 were almost equal at lower temperature (308–323 K) where influence of temperature and concentration polarization is the low. Nevertheless, with increasing the feed temperature, the vapor partial pressure of components (driving force) in the feed side and the free volume of PDMS membrane (rubbery polymer) were improved^29^,^30^, thereby significantly increasing the permeate fluxes. Thus, the influence of temperature polarization and concentration polarization were serious at high temperatures, which make such big difference between PV and GSVP process on the fluxes.

For both processes, effects of temperature and concentration on separation factor as well as the permeate concentration were investigated over the temperature range of 35–95°C at different furfural concentrations (Fig. 6). In comparison, the values of separation factor of GSVP were 1.1 to 1.77 times higher than that of PV for separating furfural/water at the same conditions, and the furfural concentration in permeate side increased by 20–120 gL^−1^ when using GSVP instead of PV for the separation. For instance, for recovering 1 wt% furfural from water at 65°C, the separation factor of GSVP was 79 while the separation factor of PV was only 43. These results showed that GSVP possessed higher selectivity for separating furfural from dilute solution than PV. This phenomenon can be explained by considering the nature of GSVP. In fact, there are two processes playing roles with respect to separation in GSVP^31^: one is the liquid-gas phase transition in gas stripping (*α_Strip_*) and the other one is vapor permeation through the membrane (*α_memb_*). Because furfural is more volatile than water, furfural concentration in vapor is easier to reach the value of vapor-liquid equilibrium (VLE). So after gas stripping, furfural concentration in vapor is close to the value of VLE, while the water concentration in vapor has a distance with the VLE date (see Supplementary Fig. S3 online). As a result, the water fluxes (Fig. 5e–f) showed in GSVP process was lower than that in PV process, and the value of *α_Strip_* is higher than that of *α_evap_* (the separation factor of VLE), thereby yielding a higher separation factor in GSVP process (*α_PV_* = α_evap_*α_memb_)^32^. In addition, concentration polarization, appearing in liquid phase instead of gas phase, is another reason for lower separation factor in PV experiments.

As shown in Fig. 6(a), in the PV process, the separation factor decreased with the increase in feed temperature. This was due to the diffusion rate of water improved more rapidly than that of furfural when temperature is increased^33^. However, in GSVP process (Fig. 6(b)), the variation of separation factor and permeate concentration with the change in temperature was different. The separation factor and permeate concentration increased with the feed temperature ranging from 35°C to 65°C and thereafter they decreased. This can be explained by considering two conflict effects: one is that the increase of the partial pressure of furfural in vapor is more rapid than that of water as the feed temperature increasing (see Supplementary Table. S1 online), which lead to a more rapid rate of increase in furfural flux than that in water flux, thereby yeilding an increase in separation factor and permeate concentration; the other one is that the free volume of membrane and mass transfer resistance decreased as temperature rose, which resulted in a reduction of separation factor and permeate concentration. Hence, the initial increase in separation factor and permeate concentration was due to the stronger increase caused by the former when compared to the reduction in membrane separation performance; and the subsequent decline indicated that the effect of the latter was more than that of the former when the feed temperature was above 65°C.

### Comparison of separation performance with literatures

Table 1 lists the comparison of separation performance of GSVP with other pervaporation performance in the literature for furfural-water separation. The furfural flux as well as separation factors of PDMS membrane in GSVP are much higher than those of PDMS membrane and HOSSM-ZIF-8-PMPS membranes in PV. Although PUU membranes in PV had higher separation factor and permeate concentration, however, due to very low total flux and furfural flux, its commercial applications are restricted. Moreover, comparing the PSI values listed in Table 1, the highest PSI 216200 (separation factor 38.6 and total flux 5750 gm^−2^h^−1^) so far was obtained by using GSVP to separate 6.0 wt% furfural/water solutions at 95°C, which was also about 2.5 times higher than that of PV. This confirmed that the GSVP is superior for the recovery of furfural. All these demonstrated that GSVP was a better alternative for furfural recovery.

### Energy consumption

**Figure 7:**
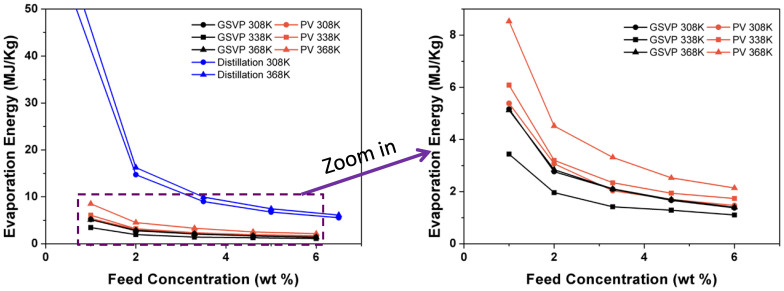
Evaporation energies of GSVP, PV and distillation required for removing furfural from the feed.

In industrial applications, the energy consumption also should be considered, besides the separation performance. Thus, the energy required to evaporate per unit of furfural in GSVP, PV and distillation (VLE date, obtained from Aspen Plus 8.0 using the NRTL model) processes were calculated and illustrated in Fig. 7, in order to make a comparison. In this study, the evaporation energy was obtained according to the following formula^36^,^37^, and the relevant physical parameters were obtained from the software, Component Plus. 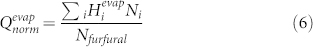
Where 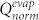 is the evaporation energy, MJkg^−1^; 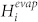 is the enthalpy of vaporization of component i, and *N_i_* is the mass flux of species i. When the feed solution and permeate mainly compose of furfural and water, the Equation (6) can be simplified as follows: 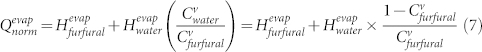
In this equation, 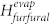 and 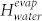 represent the heat of evaporation of furfural and water respectively. 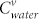 and 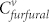 are the mass concentration of water and furfural in permeate vapor, respectively.

As Fig. 7 shows, the energy required by GSVP process is only 20% of that of distillation; and it is also reduced by 35% to 44% relative to the energy required by PV process, except at low temperature. With increasing temperature, the energy consumption for obtaining per unit furfural by GSVP decreased firstly and then increased, which is due to the variation of separation factor in GSVP. Although many factors including condensation cost, membrane exchange cost, maintenance cost, and others were not taken into account in the comparison, the evaporation energy was considered to be the main energy sink in many researchers' reports^38^,^39^,^40^. In addition, due to using gas as the feed in GSVP, coking in pipe and membrane fouling was virtually non-existent, this resulted in a reduced maintenance cost. Overall, GSVP was more energy efficient than PV and distillation.

## Conclusions

Due to the various disadvantages in using pervaporation and distillation for furfural separation, a novel separation technique, gas stripping assisted vapor permeation, was introduced in this paper. Compared to other separation techniques, GSVP has many advantages for furfural recovery. Firstly, GSVP can remove furfural polymers and recover furfural from water at the same time, which is difficult to achieve by a distillation process. Secondly, the problem of furfural polymers adhering and caking on the surface of components could be solved by using gas as the feed in GSVP process. Therefore, the corrosion and blockage, caused by coke formation, also could be avoided. Thirdly, both furfural flux and separation factor in GSVP were higher than that in PV. The highest pervaporation separation index 216200 (permeate concentration of 71.1 wt% and furfural flux of 4.09 kgm^−2^h^−1^) so far was obtained in this study by using GSVP to separate 6.0 wt% furfural/water solution at 95°C, which was also about 2.5 times higher than that of PV. Fourthly, the energy required by GSVP process is only 20% of that of distillation; and it is also reduced by 35% to 44% relative to the energy required by PV process. Finally, due to the use of gas as the feed in GSVP, the pretreatment of hydrolysate can be eliminated and the separation steps are simplified. Therefore, as demonstrated in this research, GSVP has a promising prospect for in-situ recovery of furfural, especially when coupled with the hydrolysis process or fermentation process in biorefinery industry.

## Supplementary Material

Supplementary Information Supplementary information

## Figures and Tables

**Table 1:** A comparison on furfural recovery with other reports

Process	Membrane	Feed concentration (wt%)	Feed temperature (°C)	Total flux (gm^−2^h^−1^)	Furfural flux (gm^−2^h^−1^)	Separation factor	Permeate concentration (wt%)	PSI	References
PV	PUU20[a]/HTPB[b]	2	75	46	39.6	301	86	13800	[19]
PV	PUU100/HTPB	2	75	28	26	651	93	18200	[19]
PV	Modified PUU	2	75	41.5	35.4	284	85.2	11744	[34]
PV	Silicone rubber	0.02	120	2.2	0.01	30.2	0.6	64.24	[35]
PV	HOSSM[c]-ZIF-7-PMPS	1	80	670	178.2	35.9	26.6	23383	[14]
PV	HOSSM-ZIF-8-PMPS	1	80	900	315	53.3	35	47070	[14]
VP	HOSSM-ZIF-8-PMPS	1	100	1400	422.8	42.9	30.3	58660	[14]
PV	PDMS	2	80	1538	721	43	46.9	64596	[20]
PV	PDMS	5	80	2476	1592	34	64.3	81708	[20]
PV	PDMS	2	80	2230	918.8	34.3	41.2	74259	This work
PV	PDMS	6	95	4200	2426	21.4	57.8	85680	This work
GSVP	PDMS	1	80	1404	561.6	65.4	40	90418	This work
GSVP	PDMS	2	80	1942	1064	60	54.8	114578	This work
GSVP	PDMS	6	95	5750	4090	38.6	71.1	216200	This work

[a] PUU = polyurethaneurea.

[b] HTPB = hydroxyl terminated polybutadiene.

[c] HOSSM = hierarchically ordered stainless steel mesh.
