# Supplementary material for: A novel method for furfural recovery via gas stripping assisted vapor permeation by a polydimethylsiloxane membrane
Source: Sci Rep. 2015 Mar 30;5:9428. doi: 10.1038/srep09428 (PMC4377579; doi:10.1038/srep09428)
Supplement: Supplementary Information [file srep09428-s1.pdf]

A novel method for furfural recovery via gas stripping assisted vapor permeation by a polydimethylsiloxane membrane

Song Hu, Yu Guan, Di Cai, Shufeng Li, Peiyong Qin, M. Nazmul Karim, and Tianwei Tan*

Table of Contents

Section S1. The VLE date of the binary furfural/water mixtures

Section S2. The optimization of gas stripping speed for GSVP

Section S3. Comparison of gas stripping and vapor-liquid equilibrium in the separation of furfural/water mixture

Section S4. Comparison of the saturated vapor pressure of water and furfural on various temperatures

Section S5. Comparison of PV and GSVP on the membrane performance

Section S1. The VLE date of the binary furfural/water mixtures

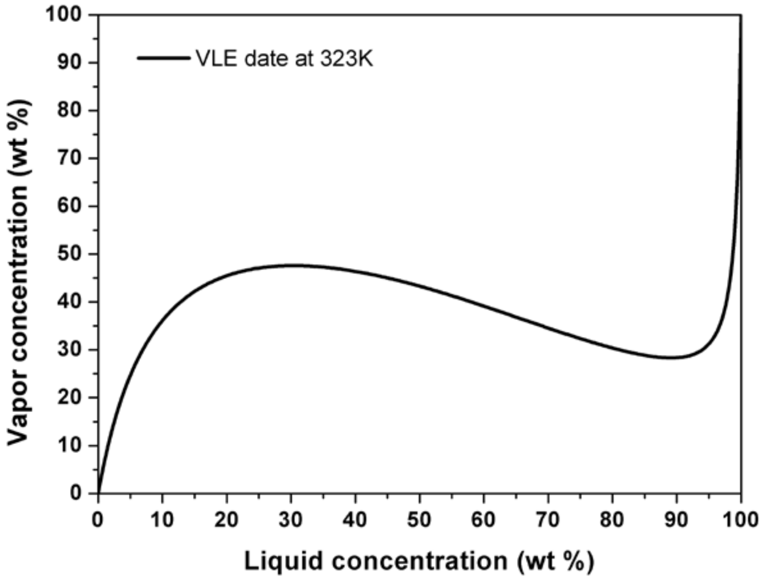

Figure S1. The diagram of vapor and liquid phase composition (mass fraction) for binary furfural/water mixtures at 323K

The dates of vapor-liquid equilibrium of furfural/water mixture were obtained from Aspen Plus 8.0 using the NRTL model, parameters for NRTL modeling were taken from the source of APV80 VLE-HOC.

Section S2. The optimization of gas stripping speed for GSVP

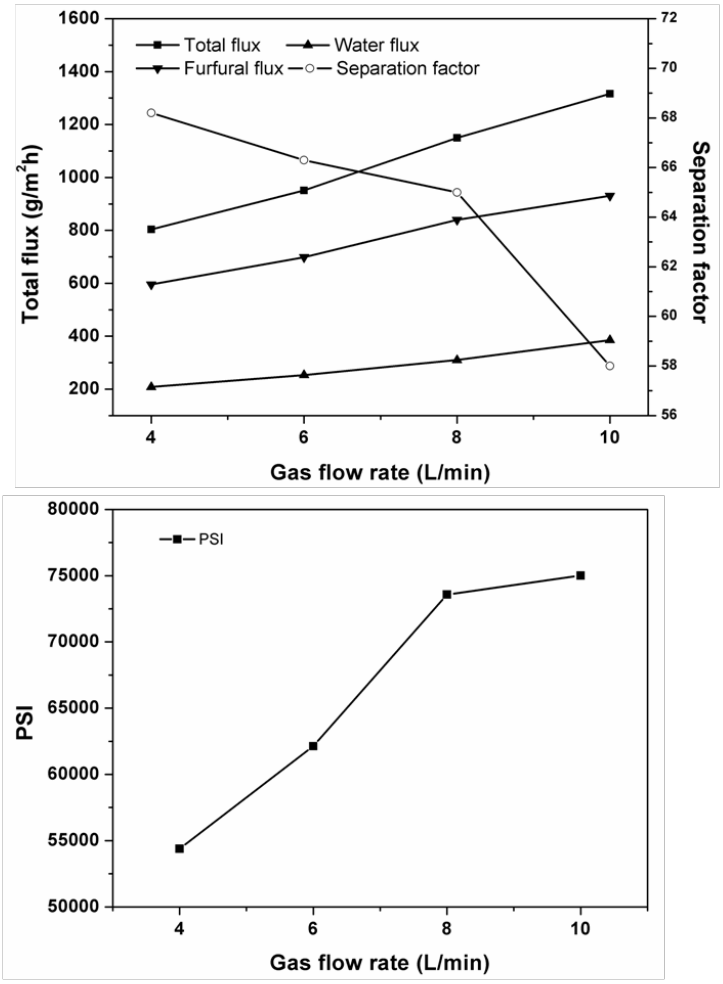

Figure S2. The effect of gas flow rate on the performance of GSVP

In the experiments, the feed solution was maintained at 60 °C with a composition of 4.0 wt % furfural. The results showed that furfural flux and water flux improved as the gas flow rate increased, with concomitant reduction in separation factor. With the gas flow rate increasing, little changes occurred in PSI value when the flow rate exceeds 8L/min.

Section S3. Comparison of gas stripping and vapor-liquid equilibrium in the separation of furfural/water mixture

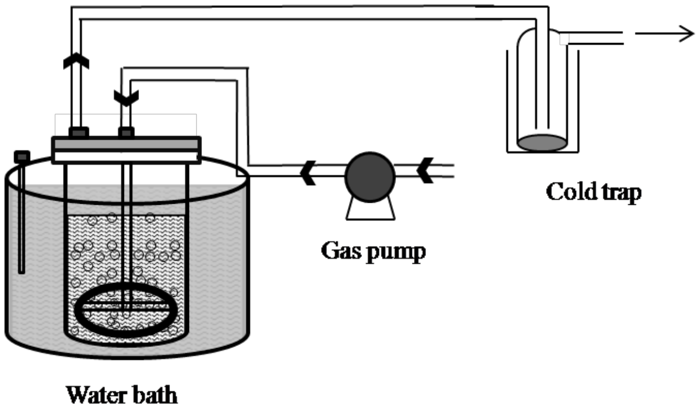

Scheme S1. Gas stripping apparatus

The experiments of gas stripping for furfural recovery were studied by using a simplified apparatus shown in Scheme S1. The feed mixture (2.5 L) with a composition of 3.3 wt % furfural was kept in the 3 L feed tanks. The gas flow rate was about 3 Lmin^{-1} and the vapor was condensed by liquid nitrogen.

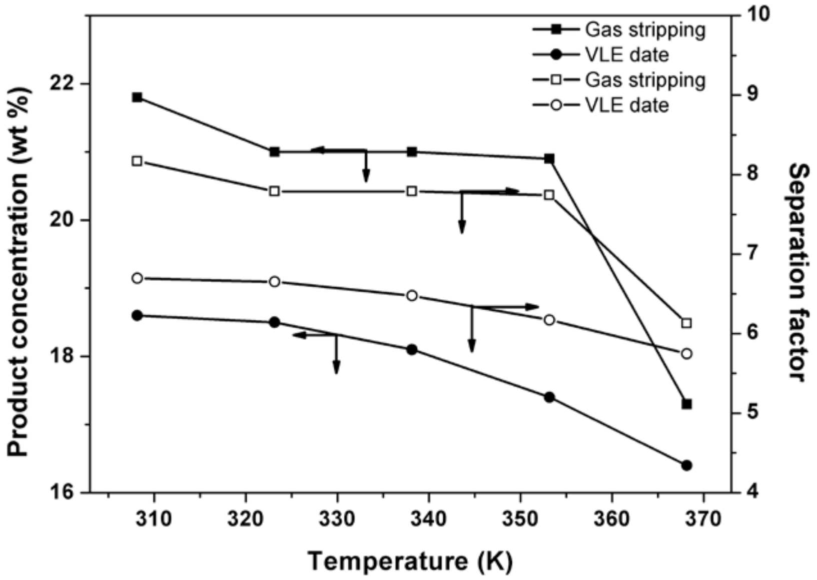

Figure S3. The comparison of gas stripping and VLE date on various temperatures

The performance of gas stripping not only depends on the vapor-liquid equilibrium, but also be affected by the mass transfer rate from liquid to vapor. As shown in Fig. S3, the product concentration after gas stripping is higher than the corresponding VLE date. Similar results were also found in removal of ABE by gas stripping in Qureshi's study.[S1] This result indicated that after gas stripping the furfural concentration (relative to water concentration) in vapor is closer to the value of VLE.

(S1) Qureshi, N. & Blaschek, H. P. Recovery of butanol from fermentation broth by gas stripping. *Renewable Energy* **22**, 557-564 (2001).

Section S4. Comparison of the saturated vapor pressure of water and furfural on various temperatures

Table S1. The saturated vapor pressure of water and furfural. (obtained from Component Plus 3.4)

Temperature (K)	Saturated vapor pressure of water $\mathbf{P_T^{water}}$ (Pa)	Saturated vapor pressure of furfural $\mathbf{P_T^{fur}}$ (Pa)	$\mathbf{P_T^{water}/P_{303K}^{water}}$	$\mathbf{P_T^{fur}/P_{303K}^{fur}}$
303	4247.9	414	1	1
318	9596	1015	2.26	2.45
333	19940	2268.6	4.69	5.48
348	38564	4683.2	9.08	11.31
363	70091.8	9025	16.50	21.80

Section S5. Comparison of PV and GSVP on the membrane performance

The comparison experiments were performed on separating 3.3 wt% furfural/water mixture at various feed temperatures. We assumed that concentration polarization didn't occurred in PV process. The membrane separation factor in both processes were calculated by the following formulas:

$$\alpha_{memb}^{PV} = \alpha_{PV}/\alpha_{evap}$$

$$\alpha_{\mathrm{memb}}^{\mathrm{GSVP}} = \alpha_{GSVP}/\alpha_{\mathrm{strip}}$$

The separation factor of VLE (α_{evap}) and gas stripping (α_{strip}) were obtained from software simulation and experiments respectively, as shown in Section S3.

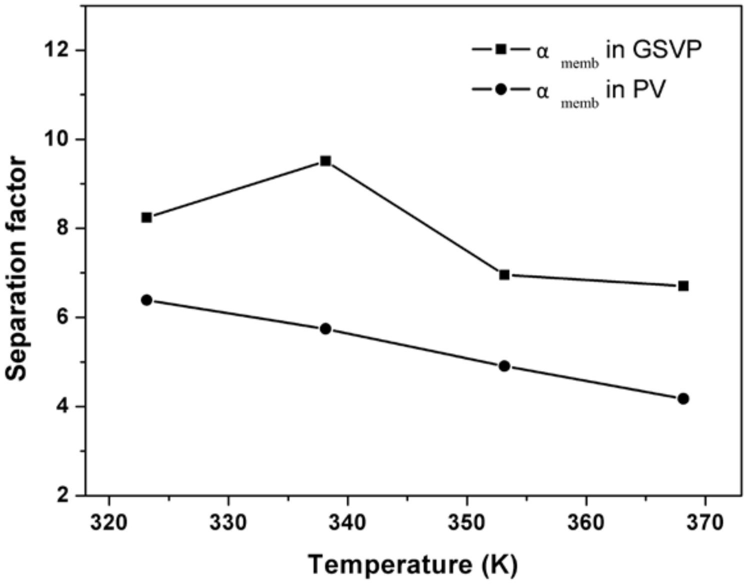

Figure S4. The comparison of PV and GSVP on the membrane separation factor

The membrane separation factor showed in GSVP is higher than that in PV. This may be caused by the fact that the effect of feed temperature on the free volume of membrane was slight in GSVP compared to that in PV.
